# Small RNAs in Outer Membrane Vesicles and Their Function in Host-Microbe Interactions

**DOI:** 10.3389/fmicb.2020.01209

**Published:** 2020-06-24

**Authors:** Sara Ahmadi Badi, Stefania Paola Bruno, Arfa Moshiri, Samira Tarashi, Seyed Davar Siadat, Andrea Masotti

**Affiliations:** ^1^Microbiology Research Center, Pasteur Institute of Iran, Tehran, Iran; ^2^Research Laboratories, Children’s Hospital Bambino Gesù-IRCCS, Rome, Italy; ^3^Gastroenterology and Liver Diseases Research Center, Research Institute for Gastroenterology and Liver Diseases, Shahid Beheshti University of Medical Sciences, Tehran, Iran

**Keywords:** gene regulation and expression, miRNA-like RNA molecules, inter-kingdom communication, small RNAs, outer membrane vesicles

## Abstract

Outer membrane vesicles (OMVs) produced by Gram-negative bacteria and their RNA content have recently received increasing attention as novel mediators of host-guest intercellular and interspecies communication. These kinds of vesicles allow not only the export of proteins, but also of DNA and other small RNAs. We previously reported that the production of bacterial vesicles could represent a way to communicate with the surrounding environment. This general mechanism, exploited by bacteria and viruses, allows conveying “messages” to the surrounding cells and tissues, thus leading to the manipulation of the host immune response. Many functions attributed to OMVs are a consequence of their high capability to survive in different environmental stressors and to enter into cells quite easily. As these properties can be exploited to advantage human health, in this review, we summarized the papers that reported the use of OMVs as modulators of cellular activities through the involvement of the RNAs contained within. We also discussed what has been done with OMVs and what remains to be discovered in order to prompt the investigations toward the complete elucidation of the role and functions of these vesicles.

## Introduction

It is well acknowledged that bacteria are able to secrete extracellular vesicles (EVs) in the surrounding environment and that these vesicles are employed to communicate with other cells (Lee et al., 2009; Deatherage and Cookson, 2012; Kim et al., 2015). Just like human cells that produce extracellular vesicles (referred as exosomes), Gram-negative bacteria release vesicles that are named outer membrane vesicles (OMVs).

Dick Hoekstra was one of the first researchers to discover vesicles from *Escherichia coli* in 1976, characterize them, and report a freeze-fracture electron micrograph of OMVs (Hoekstra et al., 1976). In the following years, vesicles were also discovered in Gram-positive bacteria (Brown et al., 2015), and novel types of outer membrane vesicles were continuously discovered (Perez-Cruz et al., 2013).

OMVs are small vesicles with a diameter ranging from 20 to 200 nm, which contain proteins, lipids, nucleic acids, and other bacterial metabolites. These vesicles have interesting properties, especially when they interact with human cells; they can deliver functional molecules to host cells, acting as nanosized delivery vectors and adjuvants in immunization strategies and likely participating in cell-to-cell communication processes (Aghasadeghi et al., 2011; Sedaghat et al., 2019). Interestingly, small RNAs (sRNAs) contained within OMVs have been considered as candidate interspecies-communication molecules due to their demonstrated capacity to modulate gene expression in multiple cell types and species (Koeppen et al., 2016; Choi et al., 2017). One of the main functions of OMVs is not only to transport but also to protect their content (in particular sRNAs) from RNAses present in the extracellular environment and to allow them to reach the host cell (Koeppen et al., 2016; Lee, 2019). Therefore, OMVs have recently received growing attention, especially for the suggested gene-regulatory roles of their sRNAs content. However, how OMVs interact with human cells, the precise mechanisms of internalization into the host cells, and the regulatory function of sRNAs remain an under-investigated area of research, especially in the context of gut microbiota field (Ahmadi Badi et al., 2017).

In order to contribute to understanding a part of the overall picture, in this review, we will briefly summarize the current findings of Gram-negative bacterial OMVs, especially by focusing on their sRNAs content, their function, and their modulatory role in the interaction with the host. Therefore, we will discuss our opinion on what should be discovered in the field of bacterial OMVs and prompt the investigations toward the complete elucidation of the roles and functions of these vesicles and their sRNAs.

## Bacterial sRNAs

In eukaryotes, small RNAs such as siRNAs and miRNAs that act as antisense regulators share common biogenesis and functional protein components (Filipowicz et al., 2005). In prokaryotes, sRNAs are structurally very heterogeneous and different in size but conserved in closely related pathogens (Bloch et al., 2017).

Many of these sRNAs have an assigned cellular function that somehow classifies them into different functional categories (Wassarman, 2002). Bacterial sRNAs have many regulatory mechanisms. Bacterial sRNAs can bind to protein targets and modify their functions such as *MicF* RNA of *E. coli* that represses the production of the outer membrane protein *OmpF* (Delihas and Forst, 2001) or *CsrB* and *CrsC* RNAs that bind the CsrA protein and reduce its activity by sequestering it from its targets (Liu et al., 1997). To regulate gene expression, bacterial sRNAs can bind to the Hfq protein (somehow similar to the RISC complex in eukaryotes) and exploit RNA base pairing to regulate the expression of target microRNAs (mRNAs). Hfq is a highly conserved and very abundant protein that has implications in a number of RNA-mediated events (Moller et al., 2002; Zhang et al., 2002). Finally, sRNAs can unmask or block the ribosome-binding site (Waters and Storz, 2009).

Bacterial sRNAs (i.e., tRNA fragments) can be internalized within extracellular vesicles, released in the surrounding environment, and transferred to other microbes and host cells (Koeppen et al., 2016; Tsatsaronis et al., 2018; Lee, 2019) as already reported by the protozoan pathogen *Trypanosoma cruzi* (Garcia-Silva et al., 2014). However, intracellular bacterial pathogens can express sRNAs that have regulatory functions similarly to miRNAs. In fact, after the infection of human THP-1 macrophage cells with the *Mycobacterium marinum*, the authors observed the production of sRNAs able to bind the host RNA-induced silencing complex (RISC) that interfered with the miRNA-mediated post-trascriptional gene regulation (Furuse et al., 2014). Also the fungal pathogen *Botrytis cinereal* has virulent sRNAs that can bind RISC and inhibit host-immunity genes (Weiberg et al., 2013). Moreover, the sRNA *PinT* produced by the intracellular pathogen *Salmonella enterica* has been demonstrated to regulate the expression of host genes and mediate the activity of invasion-associated bacterial effectors and virulence genes required for intracellular survival (Westermann et al., 2016).

Finally, periodontal pathogens have been reported to produce miRNA-sized sRNAs (msRNAs) that can be packed in OMVs and transferred into eukaryotic cells (i.e., in T lymphocytes) and induce the production of cytokines such as IL-5, IL-13, and IL-15 (Choi et al., 2017). Similarly, the *Pseudomonas aeruginosa*-derived methionine tRNA can be conveyed by OMVs into human epithelial airway cells and decreases the secretion of IL-8 (Koeppen et al., 2016).

Therefore, all of these studies emphasize the importance of microbial sRNAs as crucial communication molecules that are able to mediate host-microbe interactions.

## Biogenesis of OMVs

To our knowledge, there are four main proposed mechanisms for OMVs biogenesis (Raposo and Stoorvogel, 2013; Jan, 2017). The first model is based on the loss or rearrangement of covalent linkages between the outer membrane (OM) molecules and the underlying peptidoglycan (PG) layer allowing OM to protrude and generate OMV (Kulp and Kuehn, 2010; Kulkarni and Jagannadham, 2014). The second model hypothesizes the generation of a higher pressure inside the periplasmic space owing to the accumulation of peptidoglycan fragments or misfolded proteins in the periplasmic space that causes the OM to bulge (Haurat et al., 2015). The third suggested mechanism takes into account the enhancement of the B band lipopolysaccharide and the *Pseudomonas* quinolone signal (PQS) considered to be crucial for membrane curvature in *P. aeruginosa* and for the formation of OMVs (Kulp and Kuehn, 2010; Schertzer and Whiteley, 2012). Finally, the fourth model comes from the observation that the repression or abolishment of *VacJ/Yrb*, two genes associated with the transport and accumulation of phospholipids in the outer leaflet of OM, led to an asymmetric expansion of the outer leaflet together with a bulging of the OM and OMVs production in some Gram-negative bacteria (Roier et al., 2016).

OMVs biogenesis is modulated by growth conditions (Sarra et al., 2020) and by a different bacterial gene expression (McBroom et al., 2006; Gerding et al., 2007). Nutrients availability is another factor that could influence OMVs biogenesis. In fact, *Lysobacter* sp., and *Pseudomonas fragi* release more OMVs in response to a small or large amount of nutrients, respectively (Thompson et al., 1985; Vasilyeva et al., 2009).

In Gram-negative bacteria, the overproduction of OMVs is mediated by sRNAs through a conserved mechanism (Argaman et al., 2001; Song et al., 2008). In fact, the overexpression of *VrrA* in *Vibrio cholerae*, that has sRNAs homologs in *E. coli*, *S. enterica* serovar Typhimurium (*S.* Typhimurium), *Yersinia pestis*, and *Klebsiella pneumoniae*, increased the vesiculation by downregulating OmpA, a protein connecting OM to PG (Argaman et al., 2001; Song et al., 2008).

Different mechanisms for the selection of small RNA and miRNAs sorting into mammalian EVs have been proposed (Abels and Breakefield, 2016). These include the presence of a particular motif on the miRNA sequences (Batagov et al., 2011) that facilitate the sorting into EVs by ribonucleoproteins (i.e., hnRNPA2B1), the posttranscriptional modifications of miRNAs (i.e., 3' end uridylation), or the active involvement of crucial proteins for EVs biogenesis such as nSMase2 and AGO2.

Conversely, very little is known regarding the effect of bacterial processes and proteins involved in the selective packaging of RNA molecules into OMVs. We know that OMV biogenesis is linked to RNA packaging and sorting processes and that this process is regulated by different culture conditions (Malabirade et al., 2018). However, although during OMVs biogenesis there is a selective packaging of specific RNAs similar to that of mammalian exosomes, to our knowledge the precise sorting mechanism has never been described so far.

## Characterization of the RNA Content of Bacterial OMVs

OMVs can be typically separated from cell-free culture supernatants, liquid cultures, or body fluids (Jung et al., 2017) by differential centrifugation steps, filtration (through 0.22–0.45 μm porosity filters), and ultracentrifugation (Moon et al., 2012; Dean et al., 2019) although this isolation method does not completely remove small contaminants such as protein aggregates or membrane debris (Klimentova and Stulik, 2015). Gel filtration and density gradient ultracentrifugation are other two widely employed procedures to isolate and purify Gram-negative OMVs (Nevot et al., 2006; Chutkan et al., 2013; van de Waterbeemd et al., 2013; Antenucci et al., 2017; Kohl et al., 2018). More recently, another ultrafiltration method through a tangential flow filtration (TFF) apparatus coupled to ultracentrifugation/washing steps has been adopted to obtain a concentrated pellet of OMVs (Sarra et al., 2020).

The analysis of the inner content confirmed that these OMVs are heterogeneous nanostructures that are able to pack and transport many bacterial products and small molecules such as proteins, lipopolysaccharides (LPS), DNA fragments, and also sRNAs (Yaron et al., 2000; Renelli et al., 2004; Rumbo et al., 2011; Koeppen et al., 2016; Bitto et al., 2017; OʼDonoghue et al., 2017).

Many previous studies focused on the identification and study of OMV-associated proteins as these proteins significantly contribute to the structure and functions of Gram-negative bacterial vesicles (Kesty and Kuehn, 2004; Bai et al., 2014; Thoma et al., 2018; Veith et al., 2018). However, only recently the interest has focused also on the sRNAs content for their possible modulatory role. Table 1 reports the main papers dealing with this topic that we will discuss below. Although previous studies suggested that vesicles produced by *E. coli* and *Streptococcus mutans* contain “microRNA-like” molecules (Lee and Hong, 2012; Kang et al., 2013), the structures of these sRNAs are substantially different from eukaryotes’ microRNAs. In fact, these molecules have bulges, but not 3' overhangs, that represent two important features for mediating gene expression regulation. Ghosal and collaborators characterized the extracellular components of the OMVs of *E. coli* substrain MG1655, and they accurately described the presence of small non-coding RNAs (sRNAs; Ghosal et al., 2015). In the same year, other works emphasized the role of sRNAs and OMVs in *V. cholerae* (Sjostrom et al., 2015). In other pathogenic bacteria, such as the uropathogenic *E. coli* strain 536 and *P. aeruginosa*, independent evidence has revealed the presence of secretory bacterial sRNAs within OMVs (Blenkiron et al., 2016; Koeppen et al., 2016). More recently, also periodontal pathogens, such as *Aggregatibacter actinomycetemcomitans*, *Porphyromonas gingivalis*, and *Treponema denticola*, have been reported to secrete sRNAs in OMVs (Choi et al., 2018). Similarly, OMVs secreted by *S.* Typhimurium were characterized, revealing the presence of RNAs. The analysis of the RNA fraction showed that part of the extracellular RNA content is made by mRNAs and other non-coding RNAs that were specifically enriched in OMVs (Malabirade et al., 2018). In fact, the authors demonstrated that these sRNAs packed inside OMVs were not degraded by RNAse or proteinases as the RT-PCR of non-coding regulatory RNAs was not inhibited.

**Table 1:** Papers covering the isolation, identification and characterization of OMV-derived sRNAs.

Organism	sRNAs species identified	Followed approach	Reference
*Escherichia coli*	The authors identified sRNAs that they called “microRNA-like” molecules. These molecules are similar to eukaryotes’ miRNAs: they have bulges but not 3' overhangs.	High-throughput sequencing	Kang et al., 2013
*Streptococcus mutans*	The authors identified sRNAs that they called “microRNA-like” molecules. These molecules are similar to eukaryotes’ miRNAs: they have bulges but not 3' overhangs.	High-throughput sequencing	Lee and Hong, 2012
*Escherichia coli* K-12 substrain MG1655	The authors analyzed the extracellular RNA complement of both outer membrane vesicle (OMV)-associated and OMV-free RNAs.	High-throughput sequencing	Ghosal et al., 2015
*Vibrio cholerae* strain A1552 (O1 El Tor strain)	The authors characterized the RNA profiles of bacterial OMVs and found that RNA is among the wide variety of bacterial components associated with OMVs.	High-throughput sequencing	Sjostrom et al., 2015
*Pseudomonas aeruginosa*	Authors characterized differentially packaged sRNAs in OMVs that they transferred into human airway cells. One candidate sRNA (sRNA52320) was further studied based on its stable secondary structure and predicted mRNA targets.	High-throughput sequencing	Koeppen et al., 2016
*Escherichia coli* strain 536	The authors employed density gradient centrifugation to fractionate and characterize OMVs and they found that they carry a range of RNA species. The authors reported the first complete bacterial OMV-associated RNA profile by using RNA-sequencing of libraries derived from three different “size” RNA populations (<50 nt, 50–200 nt and 200 nt+) isolated from OMVs.	RNA-Seq (MySeq sequencing)	Blenkiron et al., 2016
*Aggregatibacter actinomycetemcomitans, Porphyromonas gingivalis*, and *Treponema denticola*	The authors identified sRNAs using deep sequencing and characterized dozens of well conserved sRNAs through bioinformatic analysis. Highly expressed microbial sRNAs were selected for further validation. They also assessed the ability of bacterial OMVs to deliver sRNAs into eukaryotic cells and identify their potential effects on human immune-related target genes (i.e., suppression of certain cytokines in Jurkat T cells).	High-throughput sequencing	Choi et al., 2018
*Salmonella enterica* serovar Typhimurium (*S.* Typhimurium)	The authors analyzed that the extracellular RNA content specifically enriched in OMVs is made by mRNAs and other non-coding RNAs. The analysis of OMV-associated RNA indicated that some sRNAs are protected by OMVs and that they can be functionally active.	High-throughput sequencing	Malabirade et al., 2018

## Uptake of OMVs by Human Cells

One of the most exciting features of OMVs is their supposed function as mediators of the communication between bacteria, the environment, and host cells through the protection of their cargo and the delivery even to distant sites (Celluzzi and Masotti, 2016; Ahmadi Badi et al., 2017). Two types of OMVs cargos have been described and they include: (i) compounds incorporated into membranes or their components and (ii) compounds contained within the OMVs lumen such as nucleic acids (i.e., DNA and RNA; Jan, 2017). It is widely accepted that several pathways promote the entry of OMVs: micropinocytosis, lipid raft-dependent or lipid raft-independent endocytosis, and clathrin‐ and caveolin-dependent entry (Canas et al., 2016; OʼDonoghue and Krachler, 2016; Turner et al., 2018). The internalization of endocytic vesicles up to 1 μm in diameter is mediated *via* micropinocytosis, whereas clathrin‐ and lipid raft-mediated endocytosis are usually implicated for the uptake of smaller vesicles.

Recent studies have demonstrated that some molecules are responsible for the entry of OMVs into host cells. Among them, LPS and the O antigen structural region are critical for OMVs entry. OMVs lacking O antigen exploit clathrin-mediated endocytosis as the main route of entry, whereas the uptake of OMVs with intact O antigen is raft-dependent (OʼDonoghue et al., 2017). Other important molecules of OMVs surface, such as the pathogen-associated molecular patterns (PAMPs), can activate TLR signaling and facilitate the entry of OMVs into the host cells. In fact, it has been demonstrated that the activation of toll-like receptor 4 (TLR4) facilitates the delivery of LPS by OMVs into the cytosol (Gu et al., 2019). Moreover, it has been observed that the OMVs membrane of *Legionella pneumophila* can fuse with eukaryotic membrane, thus mixing pathogen factors with the host cell membrane (Jager et al., 2015). Therefore, owing to the presence of different molecules, bacterial OMVs may have specific and distinct delivery routes to host cells. However, the uptake is a “multifactorial” process as it depends on many factors (for example, size, composition of the membrane and structure of its components, environmental temperature, etc.,). The complete understanding of all these parameters will help to reveal not only the biological processes that underline the guest-host communication processes, but also to devise new strategies to inhibit the action of pathogenic bacteria, facilitate the entry of OMVs for biomedical applications, and design a novel generation of powerful OMV-based engineered delivery vectors.

## Delivery of sRNAs by OMVs and Their Effect on Human Cells

OMVs from Gram-negative bacteria mediate various bacteria-bacteria interactions (Yaron et al., 2000) nutrient acquisition, biofilm development and pathogenesis (Kulp and Kuehn, 2010), antibiotic resistance (Rumbo et al., 2011), and killing of competing bacteria by directly stimulating target cells or delivering their cargos. More recently, many studies focused on the role of OMVs and their ability to enter human cells and interact with the host (Nakao et al., 2011; Pollak et al., 2012; Choi et al., 2017). Although virulence factors and other molecules can be delivered by OMVs to host cells (Kuehn and Kesty, 2005; Ellis and Kuehn, 2010), little is still known about the role (i.e., fate and function) of sRNAs contained within OMVs once delivered into host cells. As we believe that the regulatory potential of RNA molecules, including microRNAs, can have a significant impact on modulating critical biological processes in human cells, ultimately affecting human health, we focused the discussion on the most recent studies reported in the literature dealing with the delivery of OMVs containing sRNAs molecules.

In a quite recent study, Koeppen and collaborators characterized the RNA content of OMVs from *P. aeruginosa* by RNA-Seq (Koeppen et al., 2016). Among the differentially packaged sRNAs, they studied *sRNA52320*, a fragment of a methionine tRNA. This sRNA is abundant in OMVs and has a stable secondary structure. The authors studied the transfer of this sRNA to human airway cells and observed a reduction of IL-8 levels as *sRNA52320* was predicted to target MAP-kinases mRNA (Koeppen et al., 2016).

Choi and collaborators, investigated the effect of msRNAs contained in three periodontal pathogens (i.e., *A. actinomycetemcomitans*, *P. gingivalis*, and *T. denticola*) and demonstrated the uptake of OMVs by host cells (Choi et al., 2017). The authors selected one highly expressed msRNA from each periodontal pathogen and examined the levels of 16 cytokines after transfection of a synthetic msRNA oligo in Jurkat cells. They observed that the msRNAs secreted into OMVs by these periodontal pathogens were able to affect the host immune system by decreasing the expression level of IL-5, IL-13, and IL-15. Many studies in the past have linked periodontal diseases to neuroinflammation without elucidating the mechanisms of this relationship. Therefore, to explore this link, another work focused on periodontal pathogens and the role of OMV-derived RNAs, thus providing evidence about the activity of OMV-derived sRNAs in host gene regulation (Han et al., 2019). The authors demonstrated the increased production of TNF-α promoted by OMV-derived sRNAs *via* the TLR-8 and NF-κB signaling pathways. Interestingly, the intracardiac injection of OMVs in mice resulted in the successful delivery also into the brain (after BBB crossing) followed by an increased expression of TNF-α.

Although limited, these lines of evidence suggest a functional similarity of bacterial OMVs and mammalian exosomes. Similarly to exosomes that harbor miRNAs, bacterial OMVs possess sRNAs that are well protected by RNAses as demonstrated by several studies, in which OMVs RNAse treatment prior to RNA extraction did not prevent the recovery of RNA contained inside OMVs (Sjostrom et al., 2015; Koeppen et al., 2016; Choi et al., 2017). Additionally, bioinformatics approaches demonstrated that many abundant RNAs contained within OMVs are able to form stable secondary structures very similar to those of precursor miRNAs. Moreover, the sRNAs identified in *E. coli* OMVs may function through a RNA interference mechanism by pairing with complementary target genes (Masse et al., 2003).

In conclusion, it has been demonstrated that sRNAs stably incorporated within OMVs can be transferred to other bacteria or to host tissues and may play a critical regulatory role similarly to exosomal miRNAs. However, only few examples of interspecies communication *via* extracellular sRNAs appeared so far in the literature. Undoubtedly, the identification of such pathways and their species conservation strongly outline the fact that the communication through sRNAs contained into OMVs represents an important but still unexplored issue.

## Conclusions and Perspectives

Despite numerous papers demonstrated the effects of OMVs on human cells, either mediated by proteins or nucleic acids, the exact mechanisms of bacterial vesicles and their content are still largely unknown. Some of us, after the paper of Liu and coworkers (Liu et al., 2016) that reported the ability of human exosomes to regulate bacterial gene expression, suggested that bacterial vesicles might, in turn, regulate the human transcriptome and potentially induce epigenetic modifications (Celluzzi and Masotti, 2016).

**Figure 1:**
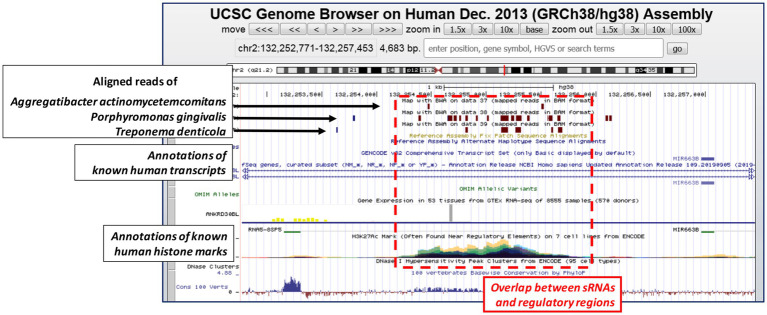
UCSC genome browser depicts one representative genomic region where the bacterial small RNAs (sRNAs) (the reads) of the three periodontal pathogens align. Brown boxes represent bacterial reads aligned (Bowtie2 with default parameters) against the human genome; the multi-view composite tracks (colored regions) reported below indicate the occurrence of ENCODE Histone Modification Track H3K27Ac found in different cell types (colored in cyan, green, yellow, red, magenta, and violet). Human genome assembly as of December 2013 (GRCh38/hg38).

As a significative example of how this concept could be valid also in other systems, we downloaded the raw data reported by Choi and colleagues (Choi et al., 2018), and we followed the same experimental bioinformatics procedures that we have already discussed in one of our previous paper (Celluzzi and Masotti, 2016). We found that the reads belonging to the three periodontal pathogens aligned against some histone mark regions of the human genome, as previously observed also for the sRNA contained in the OMVs of *E. coli* (Figure 1; Celluzzi and Masotti, 2016). These results suggested not only that these small bacterial RNAs have some similarities with regulatory regions of the human genome, but also that these molecules could function similarly to other long non-coding RNAs, already characterized in humans but still underexplored in bacteria. Therefore, we believe that these exciting findings should prompt further investigations to unravel completely the potential regulatory effects that these bacterial sRNAs might have on human cells.

Owing to the relevant regulatory potential that these bacterial sRNAs might have and the potential application of bacterial OMVs in many biomedical fields, we think that further studies should focus particularly on understanding the functions and the molecular mechanisms of these sRNAs. Moreover, we think that by studying the secondary structure of bacterial sRNAs and the similarities with other human coding and non-coding RNAs, we should be able to completely understand their function once entered into human cells. Moreover, it would be crucial to learn how to modulate important biological processes (and diseases) by engineering bacteria to produce OMVs with a specific RNA content.

## Author Contributions

SAB and SPB conceived the review and drafted the manuscript; AMo and ST wrote part of the manuscript, revised, and edited the final version; SDS contributed to the concept of the manuscript, coordinated part of the work, and wrote many parts of the manuscript; AMa contributed to the concept of the manuscript, coordinated part of the work, and finalized the final draft of the manuscript.

### Conflict of Interest

The authors declare that the research was conducted in the absence of any commercial or financial relationships that could be construed as a potential conflict of interest.
